# Mushroom allergy and anaphylaxis

**DOI:** 10.1016/j.jacig.2025.100459

**Published:** 2025-03-20

**Authors:** Öner Özdemir

**Affiliations:** Division of Allergy and Immunology, Department of Pediatrics, Research and Training Hospital of Sakarya, Sakarya University, Medical Faculty, Adapazarı, Sakarya, Turkey

To the Editor:

I read the article titled “*Agaricus bisporus* mushroom anaphylaxis: A case report and review of the literature” by Ali and Smith^1^ with great interest. This case report reviewing the literature informs us quite well about mushrooms and their species allergy. Nevertheless, clarification of a few issues related to this case report and some aspects of mushroom allergy would help to better understand the case report and the literature.

First, although the clinical picture of biphasic anaphylaxis was developed in this case report, it was not mentioned in the discussion of the case. Thirty minutes after consuming a homemade mushroom omelet with *Agaricus bisporus* spp, the patient developed rapid-onset anaphylaxis. Although she did not respond to oral antihistamines, adrenaline was not used and her condition progressed to anaphylactic shock. She recovered with adrenaline given in the arriving ambulance, but intramuscular adrenaline had to be administered in the emergency room because of a second episode of anaphylaxis. Is the progression to shock and recurrence of anaphylaxis for the second time (biphasic anaphylaxis) explained by fear of adrenaline use and/or negligence in the use of adrenaline? Or did the fact that the patient had atrial fibrillation also lead to the failure to use adrenaline and development of the clinical picture reported?

Second, Fig 1 in Ali and Smith^1^ shows the results of the skin prick test with the positive control, raw and cooked mushroom varieties (especially portobello and shiitake), and the evaluations raise the suspicion of false-positive results or irritation.^1^ The extent of positivity did not exceed the control positive/histamine wheal value in any of the skin prick tests, and the positive results obtained in the case of the samples of boiled portobello and shiitake and raw oyster mushrooms seemed doubtful even if they were said to be 4 to 5 mm in diameter. In such cases, in addition to the patient being tested, several healthy people should be tested at the same time with the same material as a control and should be found negative. Thus, the positivity obtained with the tested material would be more reliable. I wonder whether these visually unclear induration/wheal results are due to the patient having been receiving 1 mg of oral prednisolone for too long. Again, in such cases in which no clear result can be obtained, although not performed in this case, oral food challenge would be another criterion standard used to confirm the diagnosis.^2^

Third, Table I in Ali and Smith,^1^ which details the results of testing of 27 cases of mushroom allergy identified in the authors’ literature review, shows that cooked mushroom species were used for *in vivo* testing of samples from almost every patient. Only in case 14 was a boiled mushroom used for *in vivo* testing. Cooking is the preparation of any food with heating by using methods such as grilling, frying, etc. However, as a cooking method, boiling is performed in water. Should a cooking method such as boiling or frying with heat be used as a reliable standard method, or should a more sensitive method to detect mushroom allergy be used? I think that a short discussion of this issue would be good here.

Regarding a few minor points, the case discussed in by Ali and Smith^1^ here is not the first from Australia; it differs from the previously reported case in that the case patient was slightly older and developed a slightly more severe hypersensitivity reaction—a grade 3 hypotensive reaction according to Brown’s anaphylaxis grading system. In the literature, as shown in Table I in Ali and Smith,^1^ a grade 2 (moderate-grade anaphylaxis) anaphylactic reaction to *A bisporus* and *Agaricus campestris* in a 13-year-old male from Australia has been reported previously. This previously reported case also involved an adult without sensitization following occupational exposure to boiled *A bisporus* as well as exposure to other edible mushroom varieties.^3^

The sentence “Although exposure to edible fungal spores (especially in occupational settings) can lead to sensitization with rhinitis” in the second paragraph of Ali and Smith’s introduction is not clear.^1^ Here, the authors consider the possibility that those with a mushroom food allergy may be especially sensitized to mold fungal allergens owing to cross-reactive antigens, which may in turn lead to allergic rhinitis symptoms.

In conclusion, I would like to thank Ali and Smith for their case report and its results, which are accompanied by a good, high-quality literature review and inform us about mushroom food allergy.

## Disclosure statement

Disclosure of potential conflict of interest: The author declares that he has no relevant conflicts of interest.
